# Cryopreservation of Giraffe Epidydimal Spermatozoa Using Different Extenders and Cryoprotectants

**DOI:** 10.3390/ani12070857

**Published:** 2022-03-29

**Authors:** Robert Hermes, Alexis Lecu, Romain Potier, Frank Goeritz, Jessica P. Rickard, Julia Bohner, Rudy Wedlarski, Jiri Hruby, Thomas B. Hildebrandt

**Affiliations:** 1 Department Reproduction Management, Leibniz Institute for Zoo and Wildlife Research, D-10315 Berlin, Germany; goeritz@izw-berlin.de (F.G.); hildebrandt@izw-berlin.de (T.B.H.); 2 Parc Zoologique de Paris, MNHN, F-75012 Paris, France; alecu@vetosphere.com; 3 Faune Vet, F-44200 Nantes, France; rfpveto@gmail.com; 4 School of Life and Environmental Sciences, Faculty of Science, University of Sydney, Sydney, NSW 2006, Australia; jessica.rickard@sydney.edu.au; 5 Serengeti-Park Hodenhagen, D-29693 Hodenhagen, Germany; j.bohner@serengeti-park.de; 6 Bioparc de Doué-la-Fontaine, F-49700 Doué-la-Fontaine, France; rwedlarski@bioparc-zoo.fr; 7 ZOO Dvůr Králové, CZ-544 01 Dvůr Králové nad Labem, Czech Republic; jiri.hruby@zoodk.cz

**Keywords:** assisted reproduction technologies, giraffidae, freezing, epididymis, glycerol, methylformamide

## Abstract

**Simple Summary:**

Giraffe numbers have been plummeting over the last 30 years by 30–40%. As such, efforts to manage in situ and ex situ populations are increasing. Assisted reproduction techniques such as sperm cryopreservation can help preserve the genetic diversity of giraffe subspecies or enhance genetic exchange between populations. However, to date, the post-thaw motility of recovered sperm has been variable. In this study, spermatozoa were collected from the epididymides of seven giraffes to investigate whether an alternative cryoprotectant could improve sperm motility following conventional cryopreservation. For this, we compared the motility and viability of sperm prior to and after freezing in three different extenders: a commercial equine extender (BotuCrio^®^; Nidacon, Moedal, Schweden), a commercial bovine extender (Steridyl, Minitube, Tiefenbach, Germany), and an in-house “made” bovine egg yolk extender (TEY). Each was further supplemented with either glycerol or a mix of glycerol and methylformamide cryoprotectants. The results show that spermatozoa frozen with a mix of two cryoprotectants had significantly higher post-thaw motility compared to glycerol alone. Specifically, spermatozoa frozen in TEY and a mix of cryoprotectants achieved post-thaw sperm motility of 57 ± 3%. These results might serve as a blueprint for an improved protocol for giraffe sperm cryopreservation.

**Abstract:**

Giraffe numbers have plummeted over the last 30 years by 30–40%. Thus, their conservation status has been raised from least concern to vulnerable. Efforts to manage in situ and ex situ populations are increasing. Assisted reproduction techniques (ART) such as sperm cryopreservation could help preserve the genetic diversity of giraffe subspecies and, when used for artificial inseminations, enhance genetic exchange between isolated populations. However, to date, the post-thaw motility of recovered sperm has been low and inconsistent. In this study, epididymal sperm collected from the testes of giraffes (*n* = 7) was frozen in three different extenders, namely, BotuCrio, Steridyl, and test egg yolk (TEY), each supplemented with one of two different cryoprotectants (5% glycerol or a mix of 1% glycerol and 4% methylformamide) and frozen over liquid nitrogen vapor. Across all three extenders, sperm showed significantly better post-thaw results when frozen with a mix of glycerol and methylformamide compared with glycerol alone. Sperm frozen with TEY and a mix of glycerol and methylformamide achieved superior post-thaw total and progressive sperm motility of 57 ± 3% and 45 ± 3%, respectively. These results show the benefit of using alternative cryoprotectants for freezing giraffe spermatozoa and could aid in the application of ARTs for giraffe subspecies or the closely related endangered Okapi.

## 1. Introduction

In 1758, over 260 years ago, Linné first described the giraffe as one of Africa’s most charismatic species. Since then, its range has dramatically reduced due to habitat loss, habitat fragmentation, and increased human population growth. While giraffes are truly majestic creatures, they are, at the same time, an easy and rewarding target as a food source, and their skin and tail hair are popular as traditional gifts and tools. In regions of civil unrest, these features turn to their disadvantage as populations are increasingly under threat by illegal hunting [1]. It is due to these circumstances that giraffes are extinct in many parts of their former range and that the estimated number of giraffes in the wild (97,500) has declined by 30–40% over three generations. As such, the conservation status of giraffes is currently considered as a single species by the International Union of Conservation of Nature (IUCN) and has recently been raised from least concern to vulnerable [1]. 

Over the past 20 years, new genetic, morphometric, and phylogenetic research has repeatedly challenged the current giraffe taxonomy and the subdivision of the giraffe species into nine subspecies [2]. Suggestions were made to distinguish between two, six, eight, or, most recently, three or four distinct giraffe species [2,3,4,5,6]. Thus, ongoing scientific dispute on giraffe taxonomy somewhat hinders in situ or ex situ genetic management of isolated or small populations of giraffe subspecies. Assisted reproduction techniques (ARTs) such as sperm cryopreservation and artificial insemination [7] could help preserve the genetic diversity of smaller giraffe subspecies populations: for example, between captive populations of the Kordofan or the critically endangered Nubian subspecies. 

To date, data on giraffe sperm cryopreservation are scarce. A total of just five studies have reported the freezing of epididymal or ejaculated sperm using either bovine GENT extenders (Minitube; Tiefenbach, Germany) in combination with dimethylsulfoxide (DMSO) [8] or TRIS-based egg yolk extenders supplemented with 4–6% glycerol [9,10,11,12] using either conventional, freeze drying, or ultra-rapid freezing methods. From these five case studies, only two reported motile sperm of <5% and 44% post-thaw (Table 1) [8,9]. 

While both are vital studies in terms of setting an impressive benchmark for giraffe sperm cryoresistance, it is imperative that more samples are collected to reduce the variability between results and further investigate alternative cryoprotectants to improve the success of conventional freezing methods. For this, conventional freezing is by far the most straightforward method of freezing when considering the smaller amount of equipment required and widespread adoption in artificial breeding.

The successful cryopreservation of spermatozoa collected from wildlife species is vital for ARTs, in particular the establishment of biobanks to preserve genetic diversity across species [13]. Yet, depending on the intended application of frozen-thawed sperm, the minimal requirements for motility and viability post-thaw can differ greatly, depending on whether the sperm will be used for artificial insemination (AI), in vitro fertilization (IVF), or intracytoplasmatic sperm injection (ICSI). Frozen-thawed sperm for ICSI embryo production needs to be viable but not necessarily motile [11,13]. However, the critical parameter for success in the artificial insemination of a cycling female giraffe is superior sperm motility post-thaw [14]. In order to ensure that cryopreserved giraffe sperm might serve simple and advanced applications in ART, it is critical to develop freezing methods that provide both high motility and viability. There are several factors which could contribute to poor sperm quality prior to freezing, including poor animal health prior to euthanasia, time until epididymis retrieval post-mortem, and subsequent collection of spermatozoa or even collection of ejaculate from a stable floor [8,9,10,13]. However, as seen in other studies, the extender and cryoprotectant used can also have a significant impact on the quality of sperm during the freezing process. Given the giraffe’s closest domestic species are bovines, we decided to test two bovine extenders. Steridyl is a commercially available TRIS-based bovine extender (Minitube, Germany), similar to the test egg yolk (TEY) diluent which is made in house and contains 15% egg yolk. They are both used successfully in the bovine industry. Botucrio^®^ is traditionally an equine extender (Minitube, Germany), although it has had recent success preserving rhinoceros and African elephant spermatozoa [15]. Given various different concentrations of well-known cryoprotectants, glycerol, and DMSO have already been trialed in giraffe spermatozoa, it was decided to compare the effect of glycerol against a combination of glycerol and methylformamide. Methylformamide is commonly used in stallion and rhinoceros sperm cryopreservation. It is known to be more fluid than glycerol, and this aids its incorporation into the cell membrane more efficiently [15,16,17,18]. A reduction in the glycerol concentration and use of methylformamide have also previously achieved better post-thaw results in canine, stallion, and rhinoceros sperm compared to glycerol [15,19,20].

The current study took advantage of planned castrations in hybrid giraffe bulls in captive environments [21] to determine the most suitable extender and cryoprotectant for preserving sperm motility and viability post-thaw. Epididymal sperm was extracted on site immediately after castration to avoid any negative effects of the cooling and transportation of testis material on sperm quality. It was hypothesized that a combination of glycerol and methylformamide in a bovine extender would result in higher post-thaw motility, hopefully improving conventional cryopreservation protocols for the giraffe species.

## 2. Materials and Methods

### 2.1. Animals 

This study benefited from independently planned castrations or euthanasia of male giraffes in four European Association of Zoos and Aquaria (EAZA) accredited zoos. Giraffes were euthanized due to reasons unrelated to this study (*n* = 1/13 years) or castrated (*n* = 6/6–8 years) as part of the European Endangered Species Program EEP long-term goal to suspend captive management of hybrids in favor of keeping pure giraffe subspecies only [8]. Testes from 7 adult giraffes were extracted under general anesthesia. The anesthesia management and surgical technique were adjusted to the animal’s condition and veterinarian’s resources: different protocols were used, mainly based on either alpha 2 agonist (detomidine, medetomidine) combined with ketamine or butorphanol, or on highly potent opioids (etorphine, thiafentanil) [22,23,24]. The castration followed standard stallion surgery practice. The anesthesia and surgical technique differed based on the veterinarians’ personal preference. To the best of our knowledge, the choice of anesthesia protocol or surgical technique did not impact the outcome of epididymal sperm cryopreservation and was therefore a matter of personal preference of the anesthetist and surgeon, respectively.

### 2.2. Sperm Preparation and Cryopreservation

After castration, spermatozoa were extracted from both epididymides and treated identically in each of the 6 extender combinations. In brief, both cauda epididymides were immediately dissected at room temperature (RT) after castration. Each cauda epididymis was sectioned into three equal, vertical parts. To ensure that samples were not biased due to different stages of sperm maturation, sections 1, 2, and 3 of the right cauda epididymis were put into individual 37 °C, pre-warmed Petri dishes (Sarstedt, 51588 Nümbrecht Germany; 92 mm Ø; order number: 93.1646) with sections 3, 2, and 1 of the left epididymis. Epididymal parts were then minced, and 5 mL of 37 °C, pre-warmed extender was added: (1) BotuCrio (Nidacon, 43137 Mölndal, Sweden); (2) Steridyl (Minitube, 84184 Tiefenbach, Germany); and (3) test egg yolk (TEY) extender (TEY: TES 4.83 g; TRIS 1.16 g; fructose 200 mg per 100 mL of distilled water; 85 mL of the aqueous solution was mixed with 15 mL egg yolk). The Petri dish was left on the warm plate at 37.0 °C for 15 min for spermatozoa to swim out of the minced epididymis into the semen extender. Aliquots from both epididymides with the same extender were pooled, and the sperm concentration was estimated using a Neubauer hemocytometer. The aliquots were diluted to a final concentration of 150 × 10^6^ sperm/mL. After the final dilution, sperm motility and morphology and the functional integrity of the plasma membrane were assessed for each extender variant. Two different cryoprotectants were tested for each extender variant. One variant yielded a final working concentration of 5% glycerol, while the second cryoprotectant yielded a final working concentration of 1% glycerol and 4% methylformamide. The aliquots were equilibrated at RT for 10 min before they were filled in 0.5 mL straws using a micropipettor for embryo handling and a manual ball sealer (Minitube, 84184 Tiefenbach, Germany; Ref.: 19022/0002; Ref.: 13136/0100). Straws were cooled at 4 °C for 1 h and placed on a floating rack and then into a stainless-steel container filled with liquid nitrogen (LN) for freezing (Minitube, 84184 Tiefenbach, Germany; freezing unit for straws, Ref.: 15043/0636). The straws on the floating rack remained in the LN vapor, 4 cm above the LN surface for 10 min before being plunged into liquid nitrogen. The freezing rate at 4 cm above the LN was determined in advance using a temperature logger (PCE-T390, PCE Deutschland GmbH, 59872 Meschede, Germany) placed into a loaded 0.5 mL straw and then onto a floating rack in the LN bath. At 4cm above the LN, the freezing rate from +4 °C to −6 °C was −33 °C/min. The plateau phase lasted for 1:10 min:s. The freezing rate from −2 °C to −15 °C was −26 °C/min, and from −15 °C to −100 °C, it was −20 °C/min. Manual seeding was performed 60 s after freezing had started. Two straws per variant were thawed and pooled in a 37.0 °C water bath for 60 s prior to assessment.

### 2.3. Sperm Quality Assessment

Total (percentage of progressive + stationary motile sperm) and progressive (the percentage of spermatozoa which crossed at least two thirds of the field of view in a virtually progressive manner at a 200-fold magnification) sperm motility was assessed in all treatments after the initial dilution with the extender (no CP) and immediately after thawing. Subjective motility evaluations were conducted on all samples by the same experienced andrologist. For this, 7 µL aliquots of the thawed sample were placed on a pre-warmed slide, with a pre-warmed cover slip (18 mm × 18 mm), and evaluated immediately on a warm stage-equipped phase-contrast microscope using a 200 × magnification at 37 °C (Olympus C41; Olympus, Germany).

For the assessment of acrosome integrity and sperm morphology, 10 µL aliquots from the fresh diluted and frozen-thawed treatments were fixed in 40 µL Hancock’s fixative (2.9 g Tri-Natriumcitrat-2-hydrat per 100 mL distilled water; to 96 mL of the aqueous solution, 4 mL of 37% formaldehyde was added, 1:25). An amount of 10 µL of the fixed aliquots was placed on a slide with a cover slip and assessed with a phase-contrast microscope at ×1000 magnification. Acrosomes were classified as intact versus modified or reacted including completely detached acrosomes. Sperm morphology included a search for a range of abnormalities such as pyriform heads, teratoid sperm, abnormal head–tail junction, bent midpiece, broken neck, distal midpiece reflex, multiple tails, abaxial, bent, coiled or broken tails, or detached heads. Epididymal spermatozoa with a distal cytoplasmic droplet were judged as normal. 

A hyperosmotic swelling (HOS) test evaluated the function of the sperm’s membranes before and after freezing, serving as an indicator of the fertility potential of the spermatozoa [25]. For this, 20 µL aliquots of each treatment were diluted in 100 µL of sodium citrate-fructose solution (0.735 g of sodium citrate + 1.35 g fructose per 100 mL of distilled water) adjusted with water to an osmolarity of 100 mOsm and incubated for 30 min at 37 °C. From each treatment, 10 µL aliquots were placed on a slide with a cover slip and evaluated with the phase-contrast microscope. Spermatozoa were categorized as either HOS negative, i.e., cells with an unchanged tail and considered to be dead, or HOS positive, i.e., swollen cells with curved swollen tails which were considered to be viable [25]. The HOS scoring was corrected for the general occurrence of tail defects in the non-challenged samples. For acrosome integrity, morphology, and the HOS test, a total of 100 spermatozoa were evaluated.

The response to cryopreservation in each treatment was illustrated by calculating a cryoresistance ratio (CR) [12] for sperm motility, progressive motility, acrosome integrity, and membrane functional integrity:

CR = (value post-thaw/value pre-freeze) × 100

### 2.4. Statistical Analysis

Values for motility, membrane function, acrosome integrity, and normal sperm morphology were averaged across male giraffes and are reported as the mean ± SEM. The statistical significance of differences between each cryoprotectant per extender was calculated performing one-way repeated measures ANOVA with extender treatment allocated as the independent variable. A Bonferroni post-test was used to adjust the probability values if *p* < 0.05. Values of *p* < 0.05 were considered significant (GraphPad Instat, 3.0 San Diego, CA 92108, USA).

## 3. Results

**Figure 1:**
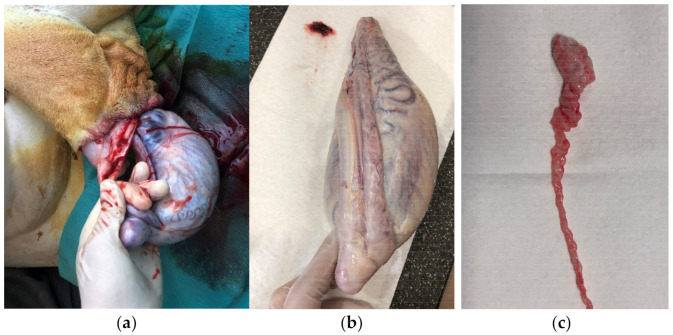
Open castration in a giraffe: (**a**) Opened processus vaginalis and protruding testis before surgical removal. (**b**) Giraffe testis, epididymis, and duct deferens. (**c**) Dissected cauda epididymis and ductus deferens.

After sperm extraction from the epididymis, sperm diluted with the TEY extender showed significantly higher pre-freeze total and progressive motility compared to the commercial extenders BotuCrio (*p* < 0.001) and Steridyl (*p* < 0.05) (Table 2, Figure 1).

In general, giraffe sperm was best preserved when frozen using the TEY extender with a mix of glycerol and methylformamide. This variant achieved the highest post-thaw total and progressive motility of 57% and 45%, respectively (Table 3), but was similar to that recorded for spermatozoa frozen in Steridyl and supplemented with glycerol and methylformamide, the commercial bovine extender (Table 3). In contrast, BotuCrio, an extender used in stallions, seemed the least suitable for the cryopreservation of giraffe sperm. Samples frozen with BotuCrio showed significantly lower post-thaw total and progressive motility compared with samples frozen with Steridyl or TEY regardless of the cryoprotectant used (*p* < 0.01).

Both commercial extenders, BotuCrio and Steridyl, showed significantly higher post-thaw total (*p* < 0.0015, *p* < 0.017) and progressive motility (*p* < 0.04, *p* < 0.013) when sperm was frozen with the mix of 1% glycerol and 4% methylformamide compared with samples frozen with 5% glycerol. Sperm frozen in the TEY extender also showed a tendency for higher post-thaw motility when frozen with the mix of cryoprotectants. Yet, this difference was not quite significant (*p* = 0.06; Table 3).

The functional integrity of the plasma membrane was significantly decreased after thawing in all extender treatments (*p* ≤ 0.03). Yet, post-thaw sperm membrane function did not differ significantly between cryoprotectants, except for semen cryopreserved in BuotoCrio with the mix of glycerol and methylformamide (*p* < 0.03).

There was a substantial decline in the normal morphology after freezing and thawing in all extender variants, especially for samples preserved in Steridyl or TEY (Table 2, Table 3 and Table 4). The decline was higher than the decline seen in HOS-positive sperm or total motility. Yet, sperm morphology and acrosome integrity prior to freezing and post-thaw did not show a significant difference between any of the extenders or cryoprotectants tested (Table 2 and Table 3).

Cryoresistance ratios of sperm motility and progressive motility showed significant differences according to the extender and cryoprotectant used. Yet, the cryoresistance ratios of sperm membrane function and acrosome integrity of ≥84 and 85, respectively, were very high across all treatments (Table 4).

## 4. Discussion

The results presented in this study demonstrate the benefits of using different cryoprotectants and conventional cryopreservation methods on the post-thaw motility of giraffe spermatozoa. Here, the comparison of frozen-thawed spermatozoa from seven bulls showed that using a commercial or custom-made bovine extender supplemented with a 1% glycerol and 4% methylformamide cryoprotectant resulted in the highest post-thaw motility of 53 ± 4% and 57 ± 3%, respectively. The custom-made bovine extender with a glycerol-alone cryoprotectant also recorded the highest progressive motility compared to the equine extender cryoprotectant variants. Interestingly, the equine extender ButoCrio supplemented with the glycerol and methylformamide cryoprotectant combination recorded the highest viability. These results signify the importance of glycerol and methylformamide as a cryoprotectant, helping to improve the survival of giraffe spermatozoa. Improved post-thaw motilities offer new opportunities for utilizing ARTs in giraffe species [7,8,9,10,12,22,26,27] and hold promise for preserving the genetics of pure-bred endangered giraffe species or the closely related Okapi.

This is the first study to report a post-thaw motility of over 44% for giraffe sperm. Indeed, of the previous studies which have investigated the cryosurvival of giraffe spermatozoa, these results comprehensively assess the impact of conventional cryopreservation protocols both before and after freezing on a number of sperm parameters. Of the papers that have reported motile spermatozoa post-thaw, our results recorded a 13% increase in motility than previously recorded [9]. Although there was no effect of treatment on the acrosome integrity of giraffe spermatozoa, the high results reported when pooled across bulls and treatments (84.2 ± 3.17) act as a benchmark for this species for future cryopreservation studies. When the cryoresistance ratios (CR) for sperm acrosome integrity and viability are also considered, these results add further evidence of the high resilience of giraffe epididymal spermatozoa to cryopreservation. Even treatments which recorded low post-thaw motility showed CRs of epididymal sperm membrane functional integrity and acrosome integrity of >84% and >85%, respectively. This suggests that epididymal giraffe sperm are robust and remain mostly viable after conventional cryopreservation, particularly when frozen with a combination of glycerol and methylformamide. Our findings are in line with case reports on ejaculated, immotile, freeze-dried giraffe sperm fertilizing mice oocytes via ICSI and on epididymal giraffe sperm which remained viable after ultra-rapid freezing [10,12]. The higher results generated in the current study compared to others, might be explained by the improved pre-freeze sperm quality and larger number of samples available in this study. Research into sperm membrane lipid composition and its cholesterol/phospholipid ratio in relation to the presence of glycerol and methylformamide might also further explain the exceedingly high cryoresistance of giraffe spermatozoa [28,29].

The combination of glycerol and methylformamide as a cryoprotectant cocktail has been investigated in canine [19], stallion [20], and rhinoceros [15] cryopreservation protocols. This protocol has been gaining momentum as a suitable cryoprotectant given it is less toxic than traditional glycerol. It could therefore be argued that perhaps giraffe species have an increased sensitivity to glycerol, and given the current cryopreservation methods have extended incubation periods in glycerol, this may aggravate any toxic effects. Glycerol cell toxicity is partly due to the high molecular weight and viscosity, resulting in a slow cell membrane penetration and thus undesirable osmotic effects and cell dehydration [30]. In contrast to glycerol, methylformamide has been reported to cause less osmotic damage to sperm because of its lower molecular weight and viscosity [15]; this allows it to permeate the plasma membrane more readily, providing a greater source of protection to the cell during freezing. Reducing the concentration of glycerol from 5% to 1% (as per conventional freezing recipes) not only reduces the toxicity of glycerol but also increases the concentration of more permeable cryoprotectants. However, these results differ from other previous studies on stallions and rhinoceroses, which showed improved post-thaw sperm motility in media containing just glycerol [15,16,17,18,19,20,28,29,31], perhaps suggesting a more species-specific role for methylformamide. In general, the mix of glycerol and methylformamide as used here in giraffes and previously in rhinoceroses [15] might prove a more suitable cryoprotectant of choice for the cryopreservation of sperm from wildlife species in which knowledge on cryoresistance of their sperm is poor or even unknown. In relation to the base extenders used, it is difficult to directly compare the difference between the TEY and commercial Steridyl extenders given the specific percentages of egg yolk and other ingredients in Steridyl are unknown. Indeed, the extenders are likely quite similar in their composition. This suggests that the success of giraffe sperm cryopreservation might be more related to the choice of cryoprotectant and its concentration, rather than the base extender ingredients themselves.

Biobanking of cryopreserved sperm, oocytes, tissue, or cell lines to safeguard the genetic diversity of endangered (sub)species evolves as a new, genuine strategy in endangered wildlife conservation [13]. In this context, improved sperm cryopreservation is immensely important for the development of ART in giraffes and the maintenance of giraffe genetics. These investigations are key for the development of protocols necessary to establish genetic, gamete, or even embryo biobanks of threatened giraffe subspecies. High-quality cryopreserved sperm might be used for AI in captive giraffes or for in vitro embryo production and embryo transfer in small populations of giraffe subspecies. The first reports on AI using frozen–thawed sperm [7], xeno-ICSI of giraffe sperm into mouse oocytes [10], or embryo retrieval in early pregnant giraffes [7,31] have set the start towards the development of ART in giraffes and building biobanks maintaining gametes and embryos as a ‘backup population’ of endangered or genetically distinct giraffe subspecies.

## 5. Conclusions

Using the conventional method of giraffe sperm cryopreservation, with a combination of glycerol and methylformamide, this study represents the first study to report a post-thaw motility of 57%. Sperm viability, and in particular acrosome integrity, also remained high. Along with the corresponding cryoresistance ratios, these results agree with previous studies which have claimed that giraffe spermatozoa are actually very resilient to the freezing process. With this in mind, perhaps the challenge is actually ensuring motility prior to freezing is acceptable, dependent on the method of semen collection, be that via epididymal harvest post-cull or castration, or ejaculated sperm recovery. Regardless, these results offer a new alternative to freezing with toxic glycerol and could see an improvement in the post-thaw motility of giraffe spermatozoa. Acting as a model for pure-bred giraffe subspecies, the use of captive-bred hybrid individuals is key to further optimizing and improving protocols for long-term genetic preservation and possibly the survival of endangered giraffe subspecies in the future.

## Figures and Tables

**Table 1:** Overview of giraffe sperm cryopreservation studies and post-thaw results.

Reference	Giraffe (*n*)	Type of Sperm	Freezing Method	Freezing Extender	Cryoprotectant	Post-Thaw Motility
Maya Soriano, 2012	1	epididymal	conventional	Gent A/B	DMSO 1%/5%/10%	<5%
Kaneko, 2014	1	Ejaculated	freeze drying	Tris-EDTA buffer	None	0
Lueders, 2015	1	Ejaculated	conventional	BIOXcell/Triladyl	Glycerol 4%/6%	not reported
Sipek, 2019	1	epididymal	conventional	Tris-fructose-citric + 20%EY	Glycerol 6%	44 %
O’Brien, 2019	2	epididymal	conventional	Tris-citric-glucose + 6%EY	Glycerol 5%	not reported
			ultra-rapid	Tris-citric-glucose + 6%EY	100 mM sucrose	not reported

EY: egg yolk.

**Table 2:** Giraffe epididymal sperm characteristics (*n* = 7) pre-freeze without cryoprotectant assessed subjectively at 37 °C. Data are pooled over *n* = 7 males ± SEM. Columns which differ in superscript indicate significant differences between treatments (*p* < 0.05).

Freezing Extender	BotuCrio	Steridyl	TEY
Total motility (%)	73.7 ± 4.4 ^a^	79.4 ± 4.1 ^a^	88.9 ± 2.4 ^b^
Progressive motility (%)	73.7 ± 4.4 ^a^	79.4 ± 4.1 ^a^	88.9 ± 2.4 ^b^
HOS positive (%)	91.0 ± 1.9 ^a^	90.1 ± 1.5 ^a^	94.4 ± 1.0 ^b^
Acrosome intact (%)	94.9 ± 1.3 ^a^	93.9 ± 1.4 ^a^	96.6 ± 0.6 ^b^
Normal morphology (%)	71.0 ± 1.8 ^a^	80.9 ± 0.8 ^b^	82.1 ± 2.4 ^b^

**Table 3:** Giraffe epididymal sperm characteristics (*n* = 7) post-thaw using glycerol and a mix of glycerol and methylformamide as cryoprotectants. Gly: 5% glycerol; Gly + MF: 1% glycerol + 4% methylformamide. Data are pooled over *n* = 7 males ± SEM. Columns which differ in superscript indicate significant differences between treatments (*p* < 0.05).

Freezing Extender	BotuCrio		Steridyl		Test Egg Yolk	
Cryoprotectant	Gly	Gly + MF	Gly	Gly + MF	Gly	Gly + MF
Total motility (%)	7.0 ± 1.9 ^a^	29.6 ± 4.1 ^b^	29.7 ± 5.6 ^b^	52.6 ± 4.2 ^c^	49.0 ± 6.0 ^c^	57.3 ± 3.0 ^c^
Progressive motility (%)	1.1 ± 0.7 ^a^	13.7 ± 5.6 ^b^	17.6 ± 6.1^b^	40.7 ± 5.1 ^c^	34.9 ± 6.6 ^c^	45.0 ± 3.3 ^c^
HOS positive (%)	76.3 ± 3.8 ^a^	86.7 ± 1.5 ^b^	75.7 ± 2.9 ^a^	79.0 ± 2.9 ^a^	84.1 ± 2.1 ^a^	82.7 ± 2.0 ^a^
Acrosome intact (%)	86.6 ± 1.2	87.3 ± 1.4	81.9 ± 3.2	82.6 ± 4.7	82.3 ± 4.6	84.4 ± 3.9
Normal morphology (%)	48.6 ± 6.1	45.4 ± 7.9	38.4 ± 5.5	34.4 ± 5.4	37.9 ± 6.8	38.3 ± 7.0

**Table 4:** Calculated cryoresistance ratio for giraffe sperm motility, progressive motility, membrane function, acrosome, and morphology. Data are pooled over *n* = 7 males ± SEM. Columns which differ in superscript indicate significant differences between treatments (*p* < 0.05).

Freezing Extender	BotuCrio		Steridyl		Test Egg Yolk	
Cryoresistance Rate	Gly	Gly + MF	Gly	Gly + MF	Gly	Gly + MF
Total motility	9.2 ± 2.4 ^a^	40.1 ± 5.0 ^b^	37.4 ± 7.0 ^b^	67.5 ± 6.8 ^c^	55.5 ± 7.0 ^c^	64.7 ± 3.8 ^c^
Progressive motility	1.5 ± 1.0 ^a^	17.5 ± 6.1 ^b^	21.8 ± 7.6 ^b^	54.4 ± 7.5 ^c^	39.5 ± 7.8 ^c^	50.8 ± 3.9 ^c^
HOS positive	83.9 ± 4.0	95.4 ± 1.4	84.1 ± 3.4	87.7 ± 3.1	89.3 ± 2.9	87.8 ± 2.8
Acrosome intact	91.5 ± 2.5	92.3 ± 2.6	87.5 ± 4.1	88.4 ± 5.6	85.3 ± 4.9	87.6 ± 4.4
Morphology	68.4 ± 8.3	64.1. ± 10.9	47.7 ± 7.1	42.7 ± 6.9	45.7 ± 8.0	45.8 ± 8.1

## Data Availability

All data are contained within the article.
